# Drought intensity and duration effects on morphological root traits vary across trait type and plant functional groups: a meta-analysis

**DOI:** 10.1186/s12862-024-02275-6

**Published:** 2024-07-04

**Authors:** Yu Sun, Christelle AM Robert, Madhav P. Thakur

**Affiliations:** 1 Institute of Ecology and Evolution, University of Bern, Bern, 3012 Switzerland; 2 Institute of Plant Sciences, University of Bern, Bern, 3013 Switzerland

**Keywords:** Root length, Root tissue density, Severe drought, Plant functional group

## Abstract

The increasing severity and frequency of drought pose serious threats to plant species worldwide. Yet, we lack a general understanding of how various intensities of droughts affect plant traits, in particular root traits. Here, using a meta-analysis of drought experiments (997 effect sizes from 76 papers), we investigate the effects of various intensities of droughts on some of the key morphological root traits. Our results show that root length, root mean diameter, and root area decline when drought is of severe or extreme intensity, whereas severe drought increases root tissue density. These patterns are most pronounced in trees compared to other plant functional groups. Moreover, the long duration of severe drought decreases root length in grasses and root mean diameter in legumes. The decline in root length and root diameter due to severe drought in trees was independent of drought duration. Our results suggest that morphological root traits respond strongly to increasing intensity of drought, which further depends on drought duration and may vary among plant functional groups. Our meta-analysis highlights the need for future studies to consider the interactive effects of drought intensity and drought duration for a better understanding of variable plant responses to drought.

**Supplementary Information:**

The online version contains supplementary material available at 10.1186/s12862-024-02275-6.

## Introduction

Increasing intensity and frequency of drought events can have strong and widespread impacts on plants, potentially with negative effects on biodiversity and ecosystem functioning [1–3]. Drought impacts on plants are often observed in their morphological traits owing to phenotypic adjustments in overcoming water stress [4–6]. Drought studies are increasingly reporting on how plants respond to prolonged periods of water scarcity, shedding light on the physiological and biochemical changes they undergo [7, 8]. These studies reveal that stress during drought can lead to reduced growth and photosynthesis and increased vulnerability to diseases in many plants [9, 10]. These findings mainly rely on aboveground plant organs [11–13], whereas recent studies highlight that belowground or root trait responses to drought are crucial in understanding overall plant responses [6, 14, 15]. Indeed, compared to aboveground plant traits such as those of leaves, root traits are more challenging to study, but they can be strong predictors of plant responses to drought, as they are in direct contact with the soil environment and are responsible for the uptake of nutrients and water [6, 16].

The impact of drought on root traits exhibits significant variation across diverse morphological characteristics and their corresponding explorative and/or exploitative strategies [17, 18]. Root morphological traits like root length and root area affect a plant’s direct access to water and nutrients in the soil [19, 20]. It has also been shown that plants can overcome drought stress by adjusting their root length, such as by elongating it to access water in deeper soils [21]. Conversely, root diameter or specific root length is lowered by drier soil conditions, favouring the plant’s ability to extract water from deeper soil layers, which is important for balancing water relations and carbon assimilation [22–24]. While thicker roots (i.e., lower specific root length) can penetrate deeper soils and are likely to transport water over long distances [25, 26], it may also depend on the hydraulic conductivity of plants that vary across different plant functional groups [27]. Many morphological root trait responses may depend on each other and on a given plant functional group, making it difficult to yield a general understanding of trait responses to drought.

Morphological root trait responses to drought could also vary among different plant functional groups, such as grasses, trees, shrubs, legumes, and forbs, due to differences in their root systems [6, 28, 29]. For example, grass roots typically are more shallow than those of other plant functional groups, and are thus well-suited for quickly absorbing water and nutrients from the topsoil, which also makes grass roots more sensitive to increasing drought stress [30]. Shrubs usually are better suited to drought due to specific features of their root traits, such as deep taproots or a high root to shoot ratio [31], especially with the hydraulic lifting ability of deep-rooted species. For instance, good lifters like *Sarcobatus vermiculatus* have a wider functional rooting depth during the long dry season, as they help to maintain hydraulic contact with the soil by virtue of their greater resistance to cavitation [32, 33]. Tree roots might not show immediate effects during the onset of drought due to their initial resistance mechanisms, such as relying on their stored water and nutrients [34, 35]. However, with prolonged and intense drought conditions, the roots could suffer significant damage, reducing the trees’ overall ability to sequester carbon [36–38]. In order to survive severe and extreme droughts, trees also develop higher root to shoot ratios and deeper root systems [39, 40], and their fine roots increase in specific root length and root tissue density under drought but decrease in their mean root diameter [41]. Yet, drought events are not instantaneous in nature, and usually take months or years to develop and impact plants [42]. Roots of some plant functional groups like trees respond variably to drought duration, for instance the specific root length decreased over the short drought duration, whereas with drought duration extending to three months, the same root trait increased [43]. Drought duration can therefore further modify the drought intensity effects on root traits, and yet, has been ignored in several drought experiments [44].

Here, using a meta-analysis, we sought to answer whether drought intensity and duration differentially affect some of the commonly measured morphological root traits across different plant functional groups. More specifically, we ask the following questions in our meta-analysis: (1) how does drought intensity affect morphological root traits, and how do they vary across various plant functional groups? (2) how does drought duration affect morphological root traits, and how do they vary across various plant functional groups?

## Methods

### Data search and selection

We searched peer-reviewed journal articles published before Nov 15th, 2021, using Web of Science. The following search term combinations were used to obtain as many articles as possible to investigate morphological root trait responses to drought: (Drought OR Extreme Drought OR Precipitation reduction) AND (Root traits OR Belowground plant trait). These search terms gave us 3246 papers, from which we first excluded all review papers. Four main criteria were set to select studies : (a) The variables selected for the experiment were wild plants and did not include agricultural plants; (b) The species composition in the selected experimental was the same in the control and drought treatment groups; (c) The data were obtained at the same temporal and spatial scales in both control and drought treatments, and at least one morphological root trait was measured; and (d) The means, standard deviations (SD), and replicates (n) of the selected variables could be directly extracted or calculated from the paper (either from the figure or from the table). We only present drought effects on a given root morphological trait when there were at least 3 independent studies reporting the response of that given trait. In total, we obtained 997 effect sizes from 76 papers based on these criteria (PRISMA diagram, Supplementary Figures S1 & S2). Morphological root traits included in our meta-analysis were root length, root mean diameter, root area, root tissue density and specific root length (Supplementary data).

As we had multiple studies on the severe drought effects on two morphological root traits (root length and root mean diameter), we expanded further analysis specific to this intensity of drought across plant functional groups. Using our database (Supplementary Data), we then classified plants into five major functional groups: trees, shrubs, forbs, grasses, and legumes. If a study provided specifics about the intensity of drought used in their experiment, we then also noted it. When such information on drought was not provided, we then used the soil moisture content data or Standardized Precipitation Index (SPI) information provided in a paper to classify drought intensity based on the drought classification from the U.S. Drought Monitor (National Drought Mitigation Center (NDMC), the U.S. Department of Agriculture (USDA) and the National Oceanic and Atmospheric Administration (NOAA) (https://droughtmonitor.unl.edu/About/AbouttheData/DroughtClassification.aspx). Using this classification, we were able to classify four kinds of drought intensity in our database: mild (or abnormally dry), moderate, severe, and extreme drought (Supplementary Figure S3). Finally, we also recorded the duration of drought treatments, which varied from 5 days to 250 days in our database. We used ImageJ software (LOCI, University of Wisconsin, U.S.A.) to extract the data from figures in case the authors did not present their data in the table.

### Data analysis

We used log response ratio (RR) to estimate the effects of various intensities of drought on morphological root traits [45]. The RR is defined as the natural log of the ratio of the mean value of a given variable in the treatment group ($\stackrel{-}{{\text{X}}_{t}}$) to that in the control group ($\stackrel{-}{{\text{X}}_{c}}$), which is used to represent the magnitude of changes in the variables.

1 $$RR=\text{ln}\frac{\stackrel{-}{{\text{X}}_{t}}}{\stackrel{-}{{\text{X}}_{c}}}$$

The mean, SD (s_t_ and s_c_ are the standard deviation value of the treatment group and control group, respectively), and n (n_t_ and n_c_ are the number of samples in the treatment group and control group, respectively), for each treatment were extracted to calculate the variance (v) from the following equation:

2 $$\text{v}=\frac{{s}_{t}^{2}}{{{n}_{t}\overline{X}}_{t}^{2}}+\frac{{s}_{c}^{2}}{{{n}_{c}\overline{X}}_{c}^{2}}$$

The reciprocal of variance ($\text{w}=\frac{1}{\text{v}}$) was considered as the weight (W) of each RR based on statistical precision. Weighted log response ratio (${\text{R}\text{R}}_{++}$) was then calculated using the following equation:

3 $${\text{R}\text{R}}_{++}=\frac{{\sum }_{i=1}^{m}{\sum }_{j=1}^{k}{{\text{w}}_{ij}\text{R}\text{R}}_{ij}}{{\sum }_{i=1}^{m}{\sum }_{j=1}^{k}{w}_{ij}}$$

**Fig. 1:**
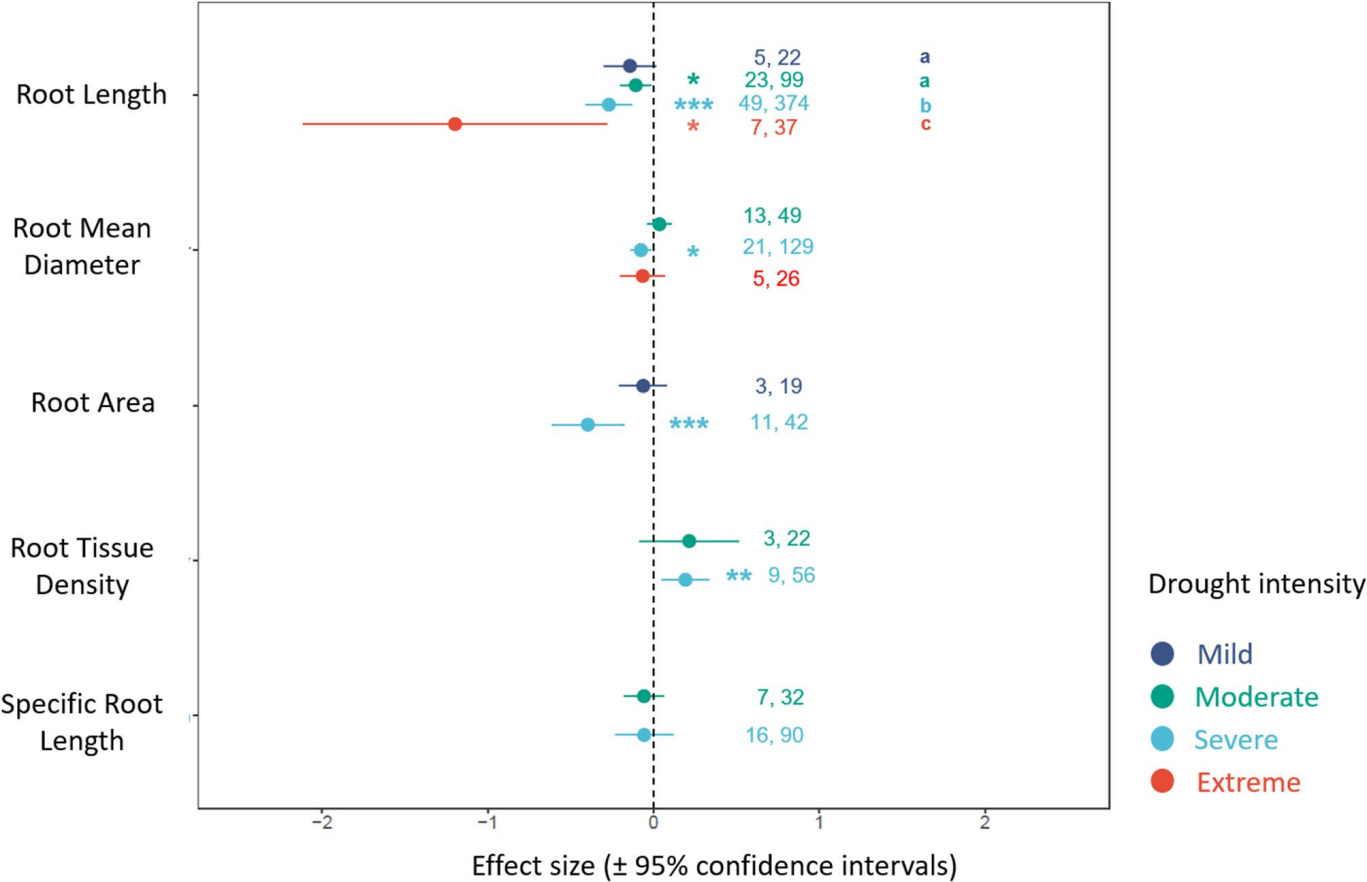
Effect sizes with 95% confidence intervals for the different intensity of droughts on morphological root traits. Effects are significant when confidence intervals do not overlap with zero (indicated by asterisks, *p-value < 0.05, **p-value < 0.01, ***p-value < 0.001). Values in figure indicate the number of studies and number of observations for the respective effect size. Different letters indicate significant differences between groups (based on Tukey post-hoc test, *p*-value < 0.05)

**Fig. 2:**
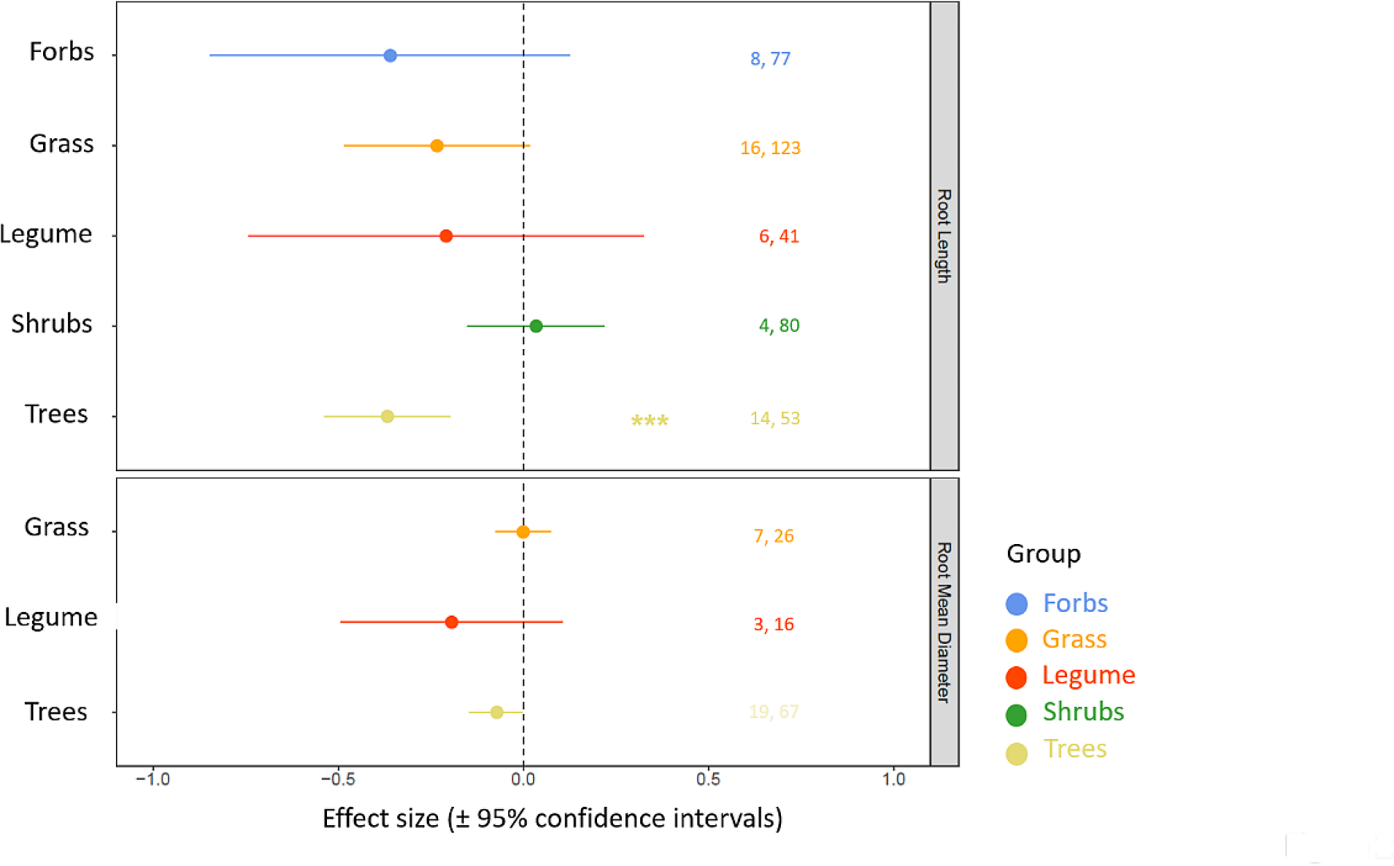
Effect sizes of severe drought (95% CI) on root length (top) and root mean diameter (bottom) across various plant functional groups. Effects are significant when confidence intervals do not overlap with zero (indicated by asterisks, ***p-value < 0.001). Values in the figure indicate the number of studies and the number of observations for the respective effect size. Effect sizes are shown for combinations of root traits and plant functional groups when at least three independent studies reported them

If 95% CI of RR_++_ for a root trait overlapped with zero, corresponding drought intensity had no significant impact on the variable. All analyses were performed in R Statistical Software [46]. The log response ratio and associated variance were calculated using the *escalc* function from the metafor package [47]. The function *rma.mv* from the metafor package was then used to conduct an inverse-variance weighted mixed-effects meta-analysis, also known as moderator analysis [47], and the restricted maximum likelihood estimation method (REML) was used for estimating the model outputs [48]. All effect sizes and their variances for drought intensity and duration were calculated in this way for morphological traits, whereas the same approach was used separately for each plant functional group to estimate corresponding effect sizes (e.g., Figs. 1 and 2; Table 1). As we had a variable number of effect sizes per study, we used each independent study as a random effect in all our meta-regression models.

**Table 1:** Drought intensity effects on morphological root traits. Effect size section includes log response ratio, 95% confidence intervals (CIs) and standard errors (SE). Test statistics include estimates of the total heterogeneity (between studies) and test statistics based on Chi- square distribution and the respective *p*-values. Significant effect sizes are indicated in bold. *df* stands for degrees of freedom

	Effect size				Test statistics			
Root traits	**Drought** **intensity**	**Estimate**	**SE**	**95% CI**	**Total heterogeneity (τ** ^**2**^ **)**	**Test for heterogeneity (Q)**	***df***	***p-*** **value**
Root Length	Mild_(5,22)_	-0.1426	0.0796	-0.2986, 0.0134	0.0300	300.1680	21	0.0731
	Moderate_(23,99)_	**-0.1078**	0.0475	**-0.2009, -0.0148**	0.0488	6641.2309	87	0.0231
	Severe_(49,374)_	**-0.2700**	0.0709	**-0.4089, -0.1310**	0.2238	63785.131	334	0.0001
	Extreme_(7,37)_	**-1.1978**	0.4681	**-2.1153, -0.2804**	1.2921	4159.8145	24	0.0105
Root Mean Diameter	Moderate_(13,49)_	0.0347	0.0374	-0.0386, 0.1080	0.0173	1394.7018	48	0.3537
	Severe_(21,129)_	-**0.0702**	0.0324	**-0.1336, -0.0068**	0.0257	33014.8176	109	0.0301
	Extreme_(5,26)_	-0.0661	0.0680	-0.1994, 0.0672	0.0240	627.0301	25	0.3310
Root Area	Mild_(3,19)_	-0.0627	0.0720	-0.2039, 0.0785	0.0137	179.7295	18	0.3845
	Severe_(11,42)_	**-0.3967**	0.1114	**-0.6150, -0.1784**	0.1217	9487.8997	41	0.0004
Root Tissue Density	Moderate_(3,22)_	0.2145	0.1518	-0.0831, 0.5121	0.0680	1221.2590	21	0.1577
	Severe_(9,56)_	**0.1915**	0.0721	**0.0501, 0.3328**	0.0603	2170.3406	55	0.0079
Specific Root Length	Moderate_(7,32)_	-0.0585	0.0601	-0.1763, 0.0594	0.0241	735.4216	31	0.3308
	Severe_(16,90)_	-0.0570	0.0882	-0.2300, 0.1159	0.1221	8042.1555	89	0.5179

Among drought intensity studies, we had the highest number of effect sizes for severe drought, and root mean diameter and root length were the most frequently measured traits in our database. These two root traits allow an understanding of how plants adapt to water scarcity [49, 50]. We accordingly tested drought duration effects for severe drought intensity on root length across five plant functional groups, whereas only across three functional groups for root mean diameter, given the data availability. In order to estimate the differences among the various plant functional groups, we used a multcomp package for Tukey’s Honest Significant Difference (HSD) tests [51].

## Results

### Drought intensity effects on morphological root traits

All drought intensity levels significantly decreased root length, except the mild drought (Fig. 1; Table 1). Root mean diameter also significantly decreased due to severe drought (Log response ratio (RR)= -0.0702, CI_95%_=-0.1336, -0.0068, Fig. 1; Table 1), but the effects of moderate and extreme drought were non-significant on this root trait (Fig. 1; Table 1). Severe drought also decreased root area (RR= -0.3967, CI_95%_=-0.6150, -0.1784, Fig. 1), with a high among-study heterogeneity (Table 1), whereas mild drought effects on root area were weak. In contrast to other morphological root trait responses, we found that root tissue density significantly increased by severe drought (RR = 0.1915, CI_95%_=0.0501, 0.3328), whereas the effect of moderate drought was non-significant (Fig. 1). Finally, specific root length showed weak responses to both moderate and severe droughts.

### Effects of severe drought on root traits across different plant functional groups

While we were not able to examine the effects of various drought intensities for different plant functional groups due to the lack of the number of studies (less than three), we were able to test the effect of one severe drought intensity across plant functional groups (Table 2). Among the different plant functional groups’ responses to severe drought, the root length of trees was most strongly affected (RR= -0.3670, CI_95%_=-0.5371,-0.1968, Fig. 2; Table 2). Our meta-analysis showed that root length of other functional groups (forbs, grass, legume and shrubs) was generally weakly affected by severe drought (Fig. 2; Table 2). Furthermore, root mean diameter of trees also showed a decreasing trend due to severe drought, whereas the effect size of severe drought on root mean diameter was non- significant in grasses and legumes (Fig. 2; Table 2).

**Table 2:** Severe drought effects on root length and root mean diameter across various plant functional groups. Effect size section includes log response ratio, 95% confidence intervals (CIs) and standard errors (SE). Test statistics include estimates of the total heterogeneity (between studies) and test statistics based on Chi- square distribution and the respective *p*-value. Significant effect sizes are indicated in bold. *df* stands for degrees for freedom

	Effect size				Test statistics					
Root traits	**Function group**_(number of studies, number of observations)_	**Estimate**	**SE**	**95% CI**	**Total heterogeneity (τ2)**	**Test for heterogeneity (Q)**			***df***	***p-value***
Root Length	Forbs_(8,77)_	-0.3593	0.2476	-0.8445, 0.1259	0.4886	3268.5399		75		0.1466
	Grass_(16,123)_	-0.2332	0.1278	-0.4837, 0.0173	0.2258	8556.1946		91		0.0680
	Legume_(6,41)_	-0.2085	0.2719	-0.7414, 0.3243	0.3679	8248.6977		30		0.4430
	Shrubs_(4,80)_	0.0345	0.0942	-0.1500, 0.2191	0.0352	2836.9742		78		0.7138
	Trees_(14,53)_	**-0.3670**	0.0005	**-0.5371,-0.1968**	0.1024	7501.2708		51		< 0.0001
Root mean Diameter	Grass_(7,26)_	-0.0004	0.0374	-0.0737, 0.0729	0.0080	707.1445		24		0.9913
	Legume_(3,16)_	-0.1936	0.1523	-0.4921, 0.1049	0.0603	2056.8582		14		0.2037
	Trees_(19,67)_	-0.0715	0.0377	-0.1453, -0.0024	0.0242	11952.3314		67		0.0579

### Effects of severe drought durations on root traits

**Fig. 3:**
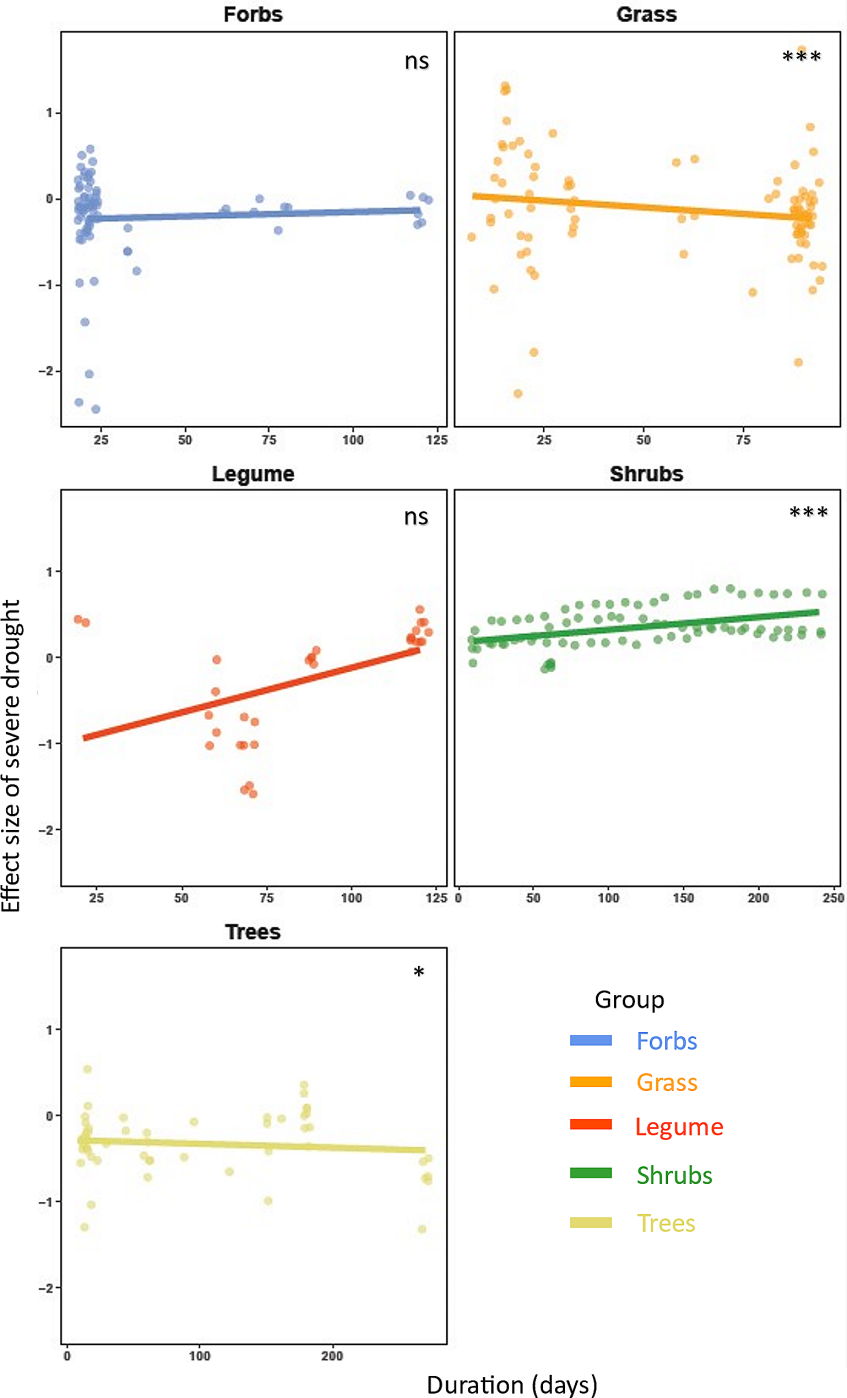
Effects of duration of severe drought on the response ratio (effect size) of root length. Each data point in the figure is a specific effect size. Linear regressions for the effect size from severe drought duration (days); Regression lines were drawn with *stat_smooth* function from the ggplot2 package [52]. ***: p-value < 0.001; *: p-value < 0.05; ns: p-value > 0.05. The detailed statistical outputs from the moderator analysis are provided in Table 3

**Fig. 4:**
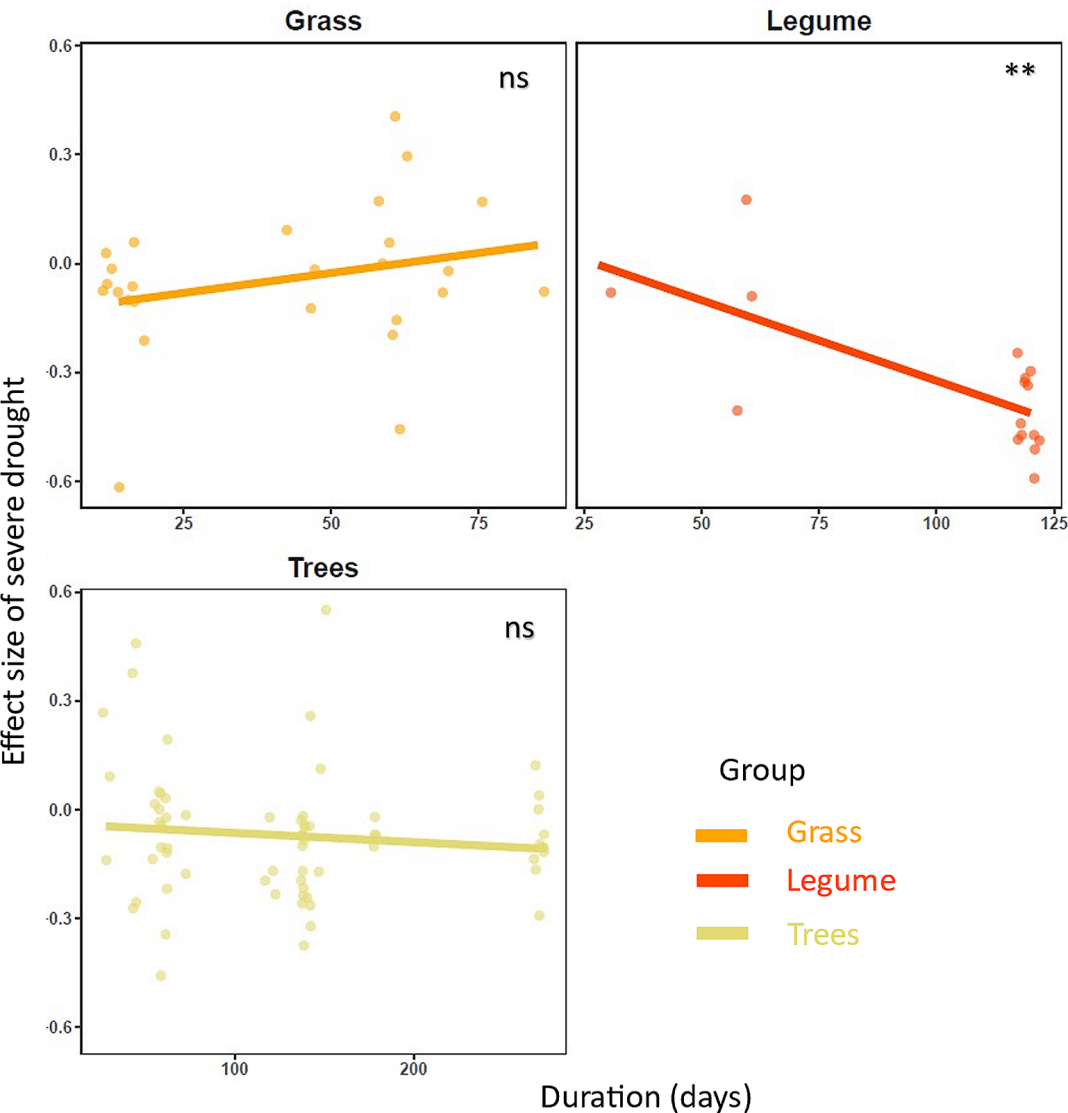
Effects of duration of severe drought on the response ratio (effect size) of root mean diameter. Each data point in the figure is a specific effect size. Linear regressions for the effect size of from severe drought duration (days); Regression lines were drawn with *stat_smooth* function from the ggplot2 package [52]. **: p-value < 0.01; ns: p-value > 0.05. The detailed statistical outputs from the moderator analysis are provided in Table 3.

Across various plant functional groups, we were able to run analyses for root length and mean root diameter responses to severe drought intensity across experimental duration of drought period ranging from 3 to 250 days. We found that the root length of grass decreased (RR= -0.0793,CI_95%_=-0.1013, -0.0573, p-value < 0.0001), shrubs increased (RR = 0.0009,CI_95%_=0.0007, 0.0011,p-value < 0.0001) and trees decreased (RR= -0.0010,CI_95%_=-0.0020, 0.000,p-value < 0.05) by the experimental duration of drought (Fig. 3). The root length of forbs and legume did not show any significant pattern with the duration of severe drought (Fig. 3; Table 3). The duration of severe drought decreased root mean diameter of legume (p-value < 0.01; Fig. 4; Table 3), whereas such responses were absent in grasses and trees (p-value > 0.05; Fig. 4; Table 3).

**Table 3:** Effects of duration of severe drought on root length and root mean diameter across different plant functional groups. Effect size section includes log response ratio, 95% CIs and SE. Test statistics include estimates of the total heterogeneity (between studies) and test statistics based on Chi- square distribution and the respective p-value. Significant effect sizes are indicated in bold. df stands for degrees for freedom

		Effect size				Test statistics			
Root trait	**Function group**	**Estimate**	**SE**	**CI**	**Total heterogeneity (τ2)**	**Test for heterogeneity (Q)**		***df***	***p-value***
Root Length	Forbs	0.0028	0.0071	-0.0110, 0.0167	0.5201	3266.3514	75		0.6905
	Grass	**-0.0793**	0.0112	**-0.10** **13, -0.0573**	6.6824	7710.0923	91		< 0.0001
	Legume	0.0003	0.0096	-0.0184, 0.0190	0.4914	8226.5985	30		0.9743
	Shrubs	**0.0009**	0.0001	**0.0007, 0.0011**	0.0263	1676.0122	78		< 0.0001
	Trees	-0.0010	0.0005	-0.0020, 0.000	0.1152	6991.6942	51		0.0438
Root mean Diameter	Grass	0.0012	0.0017	-0.0020, 0.0045	0.0091	704.4196	24		0.4636
	Legume	**-0.0055**	0.0024	**-0.0101, -0.0009**	0.0153	1942.1156	14		0.0198
	Trees	-0.0001	0.0005	-0.0012, 0.0009	0.0261	10520.9118	65		0.8296

## Discussion

Our meta-analysis shows that most morphological root traits, except for root tissue density, respond negatively across various intensities of drought (Fig. 1). The increase in root tissue density could indeed be plants’ strategy to overcome drought stress. Among plant functional groups, we found that root length and root mean diameter were most responsive to severe drought compared to all other root traits used in our meta-analysis (Fig. 1). Moreover, the duration of severe drought explained the variation in root length response to severe drought across plant functional groups (Fig. 2). While most of our results are consistent with previous meta-analyses on this topic [6, 17, 53], we further advance our current understanding of how morphological root trait responses depend not only on drought intensity, but also to some extent to drought duration, among various plant functional groups (Figs. 3 and 4).

### Effect of drought intensity on root morphological traits

A consistent decline in root length under various intensity of drought indicates a common strategy in plants to avoid or tolerate water stress (Fig. 1). Moreover, a progressively increased effect of high drought intensity on root length points inability of plants to uptake water from the soil [54], which could lead to hydraulic failure and mortality of plants particularly in trees [55]. Indeed, plants can gradually develop thicker root to support faster nutrient acquisition during drought [56, 57], which may also enhance symbiotic benefits from mycorrhizal fungi [58, 59]. Thicker roots are important in C storage [60], which is useful for maintaining osmoregulation and osmotic protection in the face of severe drought [61]. Root area is another trait related to the ability and rate of increase in total nutrient uptake by plants [62]; the ability of plants to take up water and nutrients (e.g., calcium) has been shown to be more closely related to the root area than to root weight [63]. The negative effect of severe drought on root area suggests drought induced decline in root metabolism and storage of nutrients, leading to smaller root area for exchange of resources [64].

Among all morphological root traits, only root tissue density increased in response to severe drought. This trait is often linked to plant’s ability to resist drought as a resource-conserving trait [65, 66]. Root tissue density is accordingly shown to be higher when plants are in stressful and resource-poor environments [67–69], a response that may be due to narrow, more numerous xylem vessels [56, 70], higher lignification [70], which make plants more tolerant to drought. High root tissue density reduces root turnover [71] and is often beneficial in low-nutrient environments [56], which might also allow plants to tolerate drought stress. Whether a general increase in root tissue density to severe drought would incur cost on other morphological traits merit further investigation.

### Severe drought effects across plant functional groups

Among the plant functional groups, trees responded most negatively in terms of their root length to severe drought, and the root mean diameter of the trees were also marginally significant (Fig. 2). Different from herbaceous plants, the secondary growth of trees are often more responsive to drought stress [34]. For instance, many tree species that are adapted to dry environment have a higher root: shoot ratio [39, 40], as they tend to invest more biomass into long-lasting root organs, optimizing water uptake while minimizing water loss through transpiration [72, 73]. Severe drought stress can further enhance root: shoot ratio as the biomass of fine roots in particular tends to decrease due to reduced transpiration and respiration rates; such patterns are observed both in the field [74–77], and in greenhouse experiments [78, 79] and further confirmed by meta-analyses [80, 81]. We suspect that decline in root length and root mean diameter due to severe drought most likely relates to decline in tree’s ability to invest in fine roots which are likely to trigger hydraulic failures and subsequent tree mortality. As other plant functional groups than trees did not show any significant responses to severe drought in terms of their root length and root mean diameter, it is likely that these functional groups were more plastic in their trait responses to severe drought, and it is perhaps the duration of severe drought that help us understand the variation in their responses, which we discuss below.

### Effects on root traits with increasing duration of severe drought

Our meta-analysis shows that the duration of severe drought is particularly important to understand the variation in root trait responses among plant functional groups (Figs. 3 and 4). The importance of drought duration could be understood through plants’ broad strategy to avoid and/or tolerate the water stress. Drought avoidance involves a series of adaptations that enable plants to reduce water loss and to maintain water uptake [82, 83]. In contrast, drought tolerance refers to plants’ ability to maintain essential physiological processes under water limitation [84, 85]. Drought tolerance thus involves the accumulation of osmoprotectants, antioxidants, and/or protective proteins that prevent or reduce damage caused by dehydration [86, 87].

Our results revealed that extended period of severe drought led shrubs to increase root length, although the opposite pattern was found for grasses and trees. Additionally, drought duration did not alter legumes root length significantly, but did reduce their root diameter. The different functional groups may thus favor different strategies (avoidance versus tolerance) according to their characteristics. For instance, shrubs may strongly rely on drought avoidance, as they tend to have deep root systems that can access water sources in deeper soil layers [88]. By increasing the root length when drought is prolonged, plants use a larger volume of soil and explore other unexplored areas as an effective way to increase their resource absorption capacity [89], which can be regarded as the adaptation strategy of certain plant functional groups in response to long-term drought. For instance, shrubs in a field experiment had greater root length when severe drought was prolonged [90], and they are known for their ability to adjust their root absorption surface area to acquire water that are harder to acquire otherwise [91]. The fact that this pattern is different in trees could be explained by differences in (i) resource allocation, as trees may prioritize growth aboveground over belowground [92], (ii) hydraulic strategies, as trees may enhance water uptake from existing roots instead of investing in new roots [93], (iii) drought response strategy, as trees may prioritize tolerance strategies by reducing their water needs [94], or in (iv) associations with root mycorrhizal fungi, that may support them in water acquisition [34, 95]. Grass species, that have a shorter root system and limited access to deep water sources, may use drought tolerance mechanisms to maintain essential processes. The reduction of root diameter during extended severe drought, observed in legumes only, might be a response to enhance resistance against embolism or cavitation caused by drought-induced tensions in the xylem [96]. Reducing the root diameter may further enhance hydraulic efficiency as narrower xylem vessels might improve the reliability of water transport [96, 97].

## Conclusions

Our results suggest a general pattern in many root trait responses to severe drought. Under severe drought conditions, we show a significant reduction in root length in trees, whereas responses in other plant functional groups were contingent on the duration of severe drought. More specifically, root length of grasses and trees, root mean diameter of legumes decreased with the increasing duration of severe drought, whereas the opposite pattern was found in root length of shrubs. Our study highlights the importance of considering various plant strategies to overcome drought stress, which are likely to depend not only on the intensity of drought events, but also on their duration. We recommend future studies to therefore consider the interactive effects of drought intensity and drought duration to better predict plant responses to droughts.

### Electronic supplementary material

Below is the link to the electronic supplementary material.

Supplementary Material 1

Supplementary Material 2

## Data Availability

All relevant data are within the paper and its Supporting Information files.
