# Supplementary material for: Drought intensity and duration effects on morphological root traits vary across trait type and plant functional groups: a meta-analysis
Source: BMC Ecol Evol. 2024 Jul 4;24:92. doi: 10.1186/s12862-024-02275-6 (PMC11223356; doi:10.1186/s12862-024-02275-6)
Supplement: Supplementary file 1 — Supplementary Material 1 [file 12862_2024_2275_MOESM1_ESM.docx]

**Supplemental Text 1**

List of papers from which the data were extracted for this meta-analysis.

1. Stanik, N., Lampei, C. and Rosenthal, G. (2021), Drought stress triggers differential survival and functional trait responses in the establishment of Arnica montana seedlings. Plant Biol J, 23: 1086-1096. https://doi.org/10.1111/plb.13306
2. Magda Garbowski, M. Garbowski, Danielle B. Johnston, D. B. Johnston, & Cynthia S. Brown, C. S. Brown. (2021). Cultivars of popular restoration grass developed for drought do not have higher drought resistance and do not differ in drought‐related traits from other accessions. Restoration ecology, 29, e13415. doi: 10.1111/rec.13415
3. Du, L, Liu, H, Guan, W, Li, J, Li, J. Drought affects the coordination of belowground and aboveground resource-related traits in *Solidago canadensis* in China. *Ecol Evol*. 2019; 9: 9948– 9960. <https://doi.org/10.1002/ece3.5536>
4. de Vries, F.T., Brown, C. & Stevens, C.J. (2016). Grassland species root response to drought: consequences for soil carbon and nitrogen availability. *Plant Soil* **409**, 297–312 (2016). <https://doi.org/10.1007/s11104-016-2964-4>
5. Zheng, Z; Zhang, Y; Zhang, SH; Ma, Q; Gong, DJ; Zhou, GY. (2021). Above- and belowground trait linkages of dominant species shape responses of alpine steppe composition to precipitation changes in the Tibetan Plateau, Journal of Plant Ecology, 14(4), 569–579, <https://doi.org/10.1093/jpe/rtab011>
6. Lozano, YM, Aguilar-Trigueros, CA, Flaig, IC, Rillig, MC. Root trait responses to drought are more heterogeneous than leaf trait responses. *Funct Ecol*. 2020; 34: 2224– 2235. <https://doi.org/10.1111/1365-2435.13656>
7. Ina C. Meier , Christoph Leuschner, Genotypic variation and phenotypic plasticity in the drought response of fine roots of European beech, *Tree Physiology*, Volume 28, Issue 2, February 2008, Pages 297–309, <https://doi.org/10.1093/treephys/28.2.297>
8. Shuang-Xi Zhou and others, Decoupled drought responses of fine-root versus leaf acquisitive traits among six Prunus hybrids, Journal of Plant Ecology, Volume 13, Issue 3, June 2020, Pages 304–312, <https://doi.org/10.1093/jpe/rtaa015>
9. Bristiel, P., Roumet, C., Violle, C. *et al.* Coping with drought: root trait variability within the perennial grass *Dactylis glomerata* captures a trade-off between dehydration avoidance and dehydration tolerance. *Plant Soil* **434**, 327–342 (2019). https://doi.org/10.1007/s11104-018-3854-8
10. M. Ayup, M. Ayup, X. Hao, X. Hao, Y. Chen, Y. Chen, W. Li, W. Li, & R. Su, R. Su. (0000). Changes of xylem hydraulic efficiency and native embolism of Tamarix ramosissima Ledeb. seedlings under different drought stress conditions and after rewatering. *South African journal of botany, 78*, 75-82. doi: [10.1016/j.sajb.2011.05.008](https://doi.org/10.1016/j.sajb.2011.05.008)
11. Luis Matías, L. Matías, Ignacio M. Pérez-Ramos, I. M. Pérez-Ramos, & Lorena Gómez-Aparicio, L. Gómez-Aparicio. (0000). Are northern-edge populations of cork oak more sensitive to drought than those of the southern edge?. *Environmental and experimental botany, 163*, 78-85. doi: [10.1016/j.envexpbot.2019.04.011](https://doi.org/10.1016/j.envexpbot.2019.04.011)
12. Xiang, L. S., Miao, L. F., & Yang, F. (2021). Neighbors, Drought, and Nitrogen Application Affect the Root Morphological Plasticity of *Dalbergia odorifera*. *Frontiers in plant science*, *12*, 650616. https://doi.org/10.3389/fpls.2021.650616
13. Olmo, M., Lopez-Iglesias, B. & Villar, R. Drought changes the structure and elemental composition of very fine roots in seedlings of ten woody tree species. Implications for a drier climate. *Plant Soil* **384**, 113–129 (2014). https://doi.org/10.1007/s11104-014-2178-6
14. Paganová, V., Jureková, Z., & Lichtnerová, H. (2019). The nature and way of root adaptation of juvenile woody plants Sorbus and Pyrus to drought. *Environmental monitoring and assessment*, *191*(12), 714. <https://doi.org/10.1007/s10661-019-7878-1>
15. Pirnajmedin, F., Majidi, M.M. and Gheysari, M. (2016), Survival and recovery of tall fescue genotypes: association with root characteristics and drought tolerance. Grass Forage Sci, 71: 632-640. <https://doi.org/10.1111/gfs.12231>
16. Ye, Z. Q., Wang, J. M., Wang, W. J., Zhang, T. H., & Li, J. W. (2019). Effects of root phenotypic changes on the deep rooting of *Populus euphratica* seedlings under drought stresses. *PeerJ*, *7*, e6513. <https://doi.org/10.7717/peerj.6513>
17. Ji L, Attaullah K, Wang J, Yu D, Yang Y, Yang L, Lu Z. Root Traits Determine Variation in Nonstructural Carbohydrates (NSCs) under Different Drought Intensities and Soil Substrates in Three Temperate Tree Species. Forests. 2020; 11(4):415. <https://doi.org/10.3390/f11040415>
18. Liu, Y., Li, P., Xiao, L. *et al.* Heterogeneity in short-term allocation of carbon to roots of *Pinus tabuliformis* seedlings and root respiration under drought stress. *Plant Soil* 452, 359–378 (2020). <https://doi.org/10.1007/s11104-020-04562-9>
19. Shi, Q., Yin, Y., Wang, Z., Fan, W., Guo, J., & Hua, J. (2018). Lateral Root Traits of Taxodium Hybrid ‘Zhongshanshan 406’ in Response to Drought Stress. HortScience horts, 53(4), 547-551. Retrieved Jul 20, 2023, from <https://doi.org/10.21273/HORTSCI12387-17>
20. Pirnajmedin, F., Majidi, M.M., Saeidi, G. *et al.* Genetic analysis of root and physiological traits of tall fescue in association with drought stress conditions. *Euphytica* **213**, 135 (2017). <https://doi.org/10.1007/s10681-017-1920-6>
21. Ryalls, J.M.W., Moore, B.D., Johnson, S.N. *et al.* Root responses to domestication, precipitation and silicification: weeping meadow grass simplifies and alters toughness. *Plant Soil* **427**, 291–304 (2018). <https://doi.org/10.1007/s11104-018-3650-5>
22. Wu, H., Wei, X. & Jiang, M. Intraspecific variation in seedling growth responses of a relict tree species *Euptelea pleiospermum* to precipitation manipulation along an elevation gradient. *Plant Ecol* **222**, 1297–1312 (2021). <https://doi.org/10.1007/s11258-021-01178-6>
23. Wang, C., Brunner, I., Guo, W. *et al.* Effects of long-term water reduction and nitrogen addition on fine roots and fungal hyphae in a mixed mature *Pinus koraiensis* forest. *Plant Soil* **467**, 451–463 (2021). <https://doi.org/10.1007/s11104-021-05092-8>
24. Hans Martin Hanslin, H. Martin Hanslin, Armin Bischoff, A. Bischoff, & Knut Anders Hovstad, K. Anders Hovstad. (0000). Root growth plasticity to drought in seedlings of perennial grasses. *Plant and soil, 440*, 551-568. doi: [10.1007/s11104-019-04117-7](https://doi.org/10.1007/s11104-019-04117-7)
25. Reisman-Berman, O., & Kadmon, R. (2017). Repertoire of traits in the sapling of a dwarf Mediterranean shrub confers withstanding the combined stress of drought and shade, Israel Journal of Plant Sciences, 64(1-2), 135-144. doi: <https://doi.org/10.1080/07929978.2016.1275366>
26. Rajarajan, K., & Handa, A. K. (2020). Drought stress responses in seedlings of three multipurpose agroforestry trees species of central India. *Range Management and Agroforestry*, *41*(1), 182-187.
27. Mastalerczuk, G., Borawska-Jarmułowicz, B., & Kalaji, H. M. (2017). Response of Kentucky bluegrass lawn plants to drought stress at early growth stages. *Pakistan Journal of Agricultural Sciences*, *54*(4).
28. Vega Riveros, C., Villagra, P.E. & Greco, S.A. Different root strategies of perennial native grasses under two contrasting water availability conditions: implications for their spatial distribution in desert dunes. *Plant Ecol* **221**, 633–646 (2020). <https://doi.org/10.1007/s11258-020-01038-9>
29. Padilla, F.M., Miranda, J.D., Jorquera, M.J. *et al.* Variability in amount and frequency of water supply affects roots but not growth of arid shrubs. *Plant Ecol* **204**, 261–270 (2009). <https://doi.org/10.1007/s11258-009-9589-0>
30. Fruleux A, Bonal D, Bogeat-Triboulot MB. Interactive effects of competition and water availability on above- and below-ground growth and functional traits of European beech at juvenile level Forest Ecology and Management.. 2016 Dec;382:21-30. DOI: 10.1016/j.foreco.2016.09.038.
31. Zadworny M, Mucha J, Jagodziński AM, et al. Seedling regeneration techniques affect root systems and the response of Quercus robur seedlings to water shortages Forest Ecology and Management.. 2021 Jan;479:Not Available. DOI: 10.1016/j.foreco.2020.118552.
32. DhiefAdel, AbdellaouiRaoudha, TarhouniMohamed, BelgacemAzaiez Ouled, SmitiSamira Ashi, and NeffatiMohamed. 2011. Root and aboveground growth of rhizotron-grown seedlings of three Tunisian desert *Calligonum* species under water deficit. *Canadian Journal of Soil Science*. **91**(1): 15-27. <https://doi.org/10.4141/cjss09059>
33. Mayoral C, Pardos M, Sánchez-González M, Brendel O, Pita P. Ecological implications of different water use strategies in three coexisting mediterranean tree species Forest Ecology and Management.. 2016 Dec;382:76-87. DOI: 10.1016/j.foreco.2016.10.002.
34. Zheng, H., Zhang, X., Ma, W., Song, J., Rahman, S. U., Wang, J., … & Zhang, Y. (2017). Morphological and Physiological Responses To Cyclic Drought In Two Contrasting Genotypes Of Catalpa Bungei. Environmental and Experimental Botany, (138), 77-87. <https://doi.org/10.1016/j.envexpbot.2017.02.016>
35. Yang, Y., Wang, G., Yang, L. *et al.* Effects of Drought and Warming on Biomass, Nutrient Allocation, and Oxidative Stress in *Abies fabri* in Eastern Tibetan Plateau. *J Plant Growth Regul* **32**, 298–306 (2013). <https://doi.org/10.1007/s00344-012-9298-0>
36. Huili Shi and others, Physiological and transcriptional responses of Catalpa bungei to drought stress under sufficient- and deficient-nitrogen conditions, Tree Physiology, Volume 37, Issue 11, November 2017, Pages 1457–1468, <https://doi.org/10.1093/treephys/tpx090>
37. Ovalle JF, Arellano EC, Oliet JA, Becerra P, Ginocchio R (2016). Linking nursery nutritional status and water availability post-planting under intense summer drought: the case of a South American Mediterranean tree species. iForest 9: 758-765. - doi: 10.3832/ifor1905-009
38. Lu, Y.W., Miao, X.L., Song, Q.Y. *et al.* Morphological and ecophysiological plasticity in dioecious plant *Populus tomentosa* under drought and alkaline stresses. *Photosynthetica* **56**, 1353–1364 (2018). <https://doi.org/10.1007/s11099-018-0846-0>
39. Bernat López and others, Fine roots dynamics in a Mediterranean forest: effects of drought and stem density, Tree Physiology, Volume 18, Issue 8-9, August 1998, Pages 601–606, <https://doi.org/10.1093/treephys/18.8-9.601>
40. Wang, J.P., Bughrara, S.S. Evaluation of drought tolerance for Atlas fescue, perennial ryegrass, and their progeny. *Euphytica* **164**, 113–122 (2008). <https://doi.org/10.1007/s10681-008-9669-6>
41. Fan, JW., Du, YL., Turner, N.C. *et al.* Changes in root morphology and physiology to limited phosphorus and moisture in a locally-selected cultivar and an introduced cultivar of *Medicago sativa* L. growing in alkaline soil. *Plant Soil* **392**, 215–226 (2015). <https://doi.org/10.1007/s11104-015-2454-0>
42. Naghizadeh, M., Kabiri, R., Hatami, A. *et al.* Exogenous application of melatonin mitigates the adverse effects of drought stress on morpho-physiological traits and secondary metabolites in Moldavian balm (*Dracocephalum moldavica*). *Physiol Mol Biol Plants* **25**, 881–894 (2019). <https://doi.org/10.1007/s12298-019-00674-4>
43. Mukherjee, J.R., Jones, T.A., Adler, P.B. *et al.* Drought tolerance in two perennial bunchgrasses used for restoration in the Intermountain West, USA. *Plant Ecol* **212**, 461–470 (2011). <https://doi.org/10.1007/s11258-010-9837-3>
44. Ser-Oddamba Byambadorj, Donato Chiatante, Khaulenbek Akhmadi, Janchivdorj Lunten, Batkhishig Ochirbat, Byung Bae Park, Gabriella S. Scippa, Antonio Montagnoli, Batkhuu Nyam-Osor. (2021). [The effect of different watering regimes and fertilizer addition on the growth of tree species used to afforest the semi-arid steppe of Mongolia](https://www.tandfonline.com/doi/full/10.1080/11263504.2020.1779845).*Plant Biosystems - An International Journal Dealing with all Aspects of Plant Biology* 155:4, pages 747-758.
45. Vincent Chochois and others, Variation in Adult Plant Phenotypes and Partitioning among Seed and Stem-Borne Roots across Brachypodium distachyon Accessions to Exploit in Breeding Cereals for Well-Watered and Drought Environments  , Plant Physiology, Volume 168, Issue 3, July 2015, Pages 953–967, <https://doi.org/10.1104/pp.15.00095>
46. Ma, Z, Chang, SX, Bork, EW, et al. Climate change and defoliation interact to affect root length across northern temperate grasslands. *Funct Ecol*. 2020; 34: 2611– 2621. <https://doi.org/10.1111/1365-2435.13669>
47. Delshadi, S., Ebrahimi, M., & Shirmohammadi, E. (2017). Effectiveness of plant growth promoting rhizobacteria on Bromus tomentellus Boiss seed germination, growth and nutrients uptake under drought stress. South African Journal of Botany, 113, 11-18.
48. T. Matthew Robson and others, Summer drought impedes beech seedling performance more in a sub-Mediterranean forest understory than in small gaps, Tree Physiology, Volume 29, Issue 2, February 2009, Pages 249–259, <https://doi.org/10.1093/treephys/tpn023>
49. Zegada-Lizarazu, W., Della Rocca, G., Centritto, M., Parenti, A. and Monti, A. (2018), Giant reed genotypes from temperate and arid environments show different response mechanisms to drought. Physiol Plantarum, 163: 490-501. <https://doi.org/10.1111/ppl.12701>
50. Alshameri, A., Al-Qurainy, F., Khan, S., Nadeem, M., Gaafar, A. R., Alameri, A., ... & Ashraf, M. (2019). Morpho-physiological responses of guar [Cyamopsis tetragonoloba (L.) Taub.] to multiple stresses of drought, heat and salinity. *Pak. J. Bot*, *51*(3), 817-822.
51. DELİGÖZ, AYŞE and BAYAR, ESRA (2018) "Drought stress responses of seedlings of two oak species (Quercus cerris and Quercus robur)," Turkish Journal of Agriculture and Forestry: Vol. 42: No. 2, Article 6. <https://doi.org/10.3906/tar-1709-29>
52. Lu, Y., Zhang, B., Li, L., Zeng, F. & Li, X. (2021) Negative effects of long-term exposure to salinity, drought, and combined stresses on halophyte *Halogeton glomeratus*. *Physiologia Plantarum*, 173( 4), 2307– 2322. Available from: <https://doi.org/10.1111/ppl.13581>
53. Shafqat, W., M.J. Jaskani, R. Maqbool, A.S. Khan and Z. Ali, 2019. Evaluation of citrus rootstocks against drought, heat and combined stress based on growth and photosynthetic pigments. Intl. J. Agric. Biol., 22: 1001‒1009. DOI: 10.17957/IJAB/15.1160
54. Paganová V, Hus M, Jureková Z. Physiological Performance of *Pyrus pyraster* L. (Burgsd.) and *Sorbus torminalis* (L.) Crantz Seedlings under Drought Treatment. Plants. 2020; 9(11):1496. <https://doi.org/10.3390/plants9111496>
55. Somayeh Delshadi, Mahdieh Ebrahimi & Ebrahimi Shirmohammadi (2017) Influence of plant-growth-promoting bacteria on germination, growth and nutrients’ uptake of *Onobrychis sativa* L. under drought stress, Journal of Plant Interactions, 12:1, 200-208, DOI: [10.1080/17429145.2017.1316527](https://doi.org/10.1080/17429145.2017.1316527)
56. Yu, J., Liu, M., Yang, Z., & Huang, B. (2015). Growth and Physiological Factors Involved in Interspecific Variations in Drought Tolerance and Postdrought Recovery in Warm- and Cool-season Turfgrass Species. Journal of the American Society for Horticultural Science J. Amer. Soc. Hort. Sci., 140(5), 459-465. Retrieved Jul 20, 2023, from <https://doi.org/10.21273/JASHS.140.5.459>
57. Marinoni, L.R., Richard, G.A., Bustos, D. *et al.* Differential response of *Trichloris* ecotypes from different habitats to drought and salt stress. *Theor. Exp. Plant Physiol.* **32**, 213–229 (2020). <https://doi.org/10.1007/s40626-020-00182-x>
58. Leyre Corcuera and others, Differences in hydraulic architecture between mesic and xeric Pinus pinaster populations at the seedling stage, Tree Physiology, Volume 32, Issue 12, December 2012, Pages 1442–1457, <https://doi.org/10.1093/treephys/tps103>
59. Tamayo-Chim, M., Reyes-García, C. & Orellana, R. A combination of forage species with different responses to drought can increase year-round productivity in seasonally dry silvopastoral systems. *Agroforest Syst* **84**, 287–297 (2012). <https://doi.org/10.1007/s10457-011-9470-8>
60. Andrea Bueno and others, Responses of native and invasive woody seedlings to combined competition and drought are species-specific, Tree Physiology, Volume 41, Issue 3, March 2021, Pages 343–357, <https://doi.org/10.1093/treephys/tpaa134>
61. Serrano, M.S., Perez, F.J., & Gómez‐Aparicio, L. (2021). Disentangling the interactive effects of climate change and Phytophthora cinnamomi on coexisting Mediterranean tree species. Agricultural and Forest Meteorology, 108295.
62. Alam, H, Khattak, JZK, Ksiksi, TS, et al. Negative impact of long-term exposure of salinity and drought stress on native *Tetraena mandavillei* L. *Physiologia Plantarum*. 2021; 172: 1336– 1351. <https://doi.org/10.1111/ppl.13273>
63. Badri, M., Toumi, G., Mahfoudh, S., Hessini, K., Abdelguerfi-Laouar, M., Abdelguerfi, A., Aouani, M.E., Abdelly, C. and Djébali, N. (2016), Diversity of Response to Drought in a Collection of Lines of *Medicago truncatula*, *M. ciliaris,* and *M. polymorpha*. Crop Science, 56: 3125-3132. <https://doi.org/10.2135/cropsci2016.04.0224>
64. Johannes Heinze and others, Soil temperature modifies effects of soil biota on plant growth, Journal of Plant Ecology, Volume 10, Issue 5, October 2017, Pages 808–821, <https://doi.org/10.1093/jpe/rtw097>
65. Gao, R., Shi, X. & Wang, J.R. Comparative studies of the response of larch and birch seedlings from two origins to water deficit. *N.Z. j. of For. Sci.* **47**, 14 (2017). <https://doi.org/10.1186/s40490-017-0095-1>
66. EspinozaS. E., MagniC. R., MartínezV. A., & IvkovićM. (2013). The effect of water availability on plastic responses and biomass allocation in early growth traits of Pinus radiata D. Don. *Forest Systems*, *22*(1), 3-14. <https://doi.org/10.5424/fs/2013221-02084>
67. Mohammadi, H., Dashi, R., Farzaneh, M. *et al.* Effects of beneficial root pseudomonas on morphological, physiological, and phytochemical characteristics of *Satureja hortensis* (Lamiaceae) under water stress. *Braz. J. Bot* **40**, 41–48 (2017). <https://doi.org/10.1007/s40415-016-0319-2>
68. Ge, Y., Chang, J., Li, WC. *et al.* Effect of Soil Moisture on the Gas Exchange of *Changium smyrnioides* and *Anthriscus sylvestris* . *Biologia Plantarum* **47**, 605–608 (2003). <https://doi.org/10.1023/B:BIOP.0000041071.58437.e6>
69. Sultan, S.E., Barton, K. and Wilczek, A.M. (2009), Contrasting patterns of transgenerational plasticity in ecologically distinct congeners. Ecology, 90: 1831-1839. <https://doi.org/10.1890/08-1064.1>
70. Robakowski P, Wyka TP, Kowalkowski W, Barzdajn W, Pers-Kamczyc E, Jankowski A, Politycka B. Practical Implications of Different Phenotypic and Molecular Responses of Evergreen Conifer and Broadleaf Deciduous Forest Tree Species to Regulated Water Deficit in a Container Nursery. Forests. 2020; 11(9):1011. <https://doi.org/10.3390/f11091011>
71. Celikcan, F., Koçak, M.Z., & Kulak, M. (2021). Vermicompost applications on growth, nutrition uptake and secondary metabolites of Ocimum basilicum L. under water stress: A comprehensive analysis. Industrial Crops and Products, 171, 113973.
72. Kwak, M., Lee, S., & Woo, S.Y. (2011). Growth and anatomical characteristics of different water and light intensities on cork oak (Quercus suber L.) seedlings. African Journal of Biotechnology, 10, 10964-10979.
73. Németh-Zámbori, É., Pluhár, Z., Szabó, K., Malekzadeh, M., Radácsi, P., Inotai, K., Komáromi, B., & Seidler-Lozykowska, K. (2016). Effect of water supply on growth and polyphenols of lemon balm (Melissa officinalis L.) and thyme (Thymus vulgaris L.), Acta Biologica Hungarica Acta Biologica Hungarica, 67(1), 64-74. doi: <https://doi.org/10.1556/018.67.2016.1.5>
74. Priyanka Dhar, P. Dhar, Debapam Ojha, D. Ojha, C.S. Kar, C. Kar, & Jiban Mitra, J. Mitra. (0000). Differential response of tossa jute (Corchorus olitorius) submitted to water deficit stress. *Industrial crops and products, 112*, 141-150. doi: [10.1016/j.indcrop.2017.10.044](https://doi.org/10.1016/j.indcrop.2017.10.044)
75. Pearson, M., Saarinen, M., Nummelin, L., Heiskanen, J., Roitto, M., Sarjala, T., & Laine, J. (2013). Tolerance of peat-grown Scots pine seedlings to waterlogging and drought: Morphological, physiological, and metabolic responses to stress. Forest Ecology and Management, 307, 43-53. <https://doi.org/10.1016/j.foreco.2013.07.007>
76. Chieppa, J., Nielsen, U.N., Tissue, D.T. *et al.* Drought and phosphorus affect productivity of a mesic grassland via shifts in root traits of dominant species. *Plant Soil* **444**, 457–473 (2019). https://doi.org/10.1007/s11104-019-04290-9
