# Supplementary material for: Drought intensity and duration effects on morphological root traits vary across trait type and plant functional groups: a meta-analysis
Source: BMC Ecol Evol. 2024 Jul 4;24:92. doi: 10.1186/s12862-024-02275-6 (PMC11223356; doi:10.1186/s12862-024-02275-6)
Supplement: Supplementary file 2 — Supplementary Material 2 [file 12862_2024_2275_MOESM2_ESM.docx]

Records identified from web of science:

Databases (n >5000)

**Identification**

Records excluded because they did not fit with the criteria used in the study.

(n=3113)

Records screened based on article title and abstract.

(n = 3246)

Reports sought for retrieval.

(n = 133)

Full-text articles excluded because they did not fit with the criteria used in the study.

(n=26)

**Screening**

Full-text articles excluded because the number of studies less than three have been deleted.

(n=31)

Reports assessed for eligibility.

(n =107)

Studies included in quantitative synthesis(meta-analysis) review.

(n = 76)

**Included**

*From:*  Page MJ, McKenzie JE, Bossuyt PM, Boutron I, Hoffmann TC, Mulrow CD, et al. The PRISMA 2020 statement: an updated guideline for reporting systematic reviews. BMJ 2021;372: n71. doi: 10.1136/bmj. n71

**Fig S1. Flowchart diagram of the process of obtaining literature data to build a database for this study.**


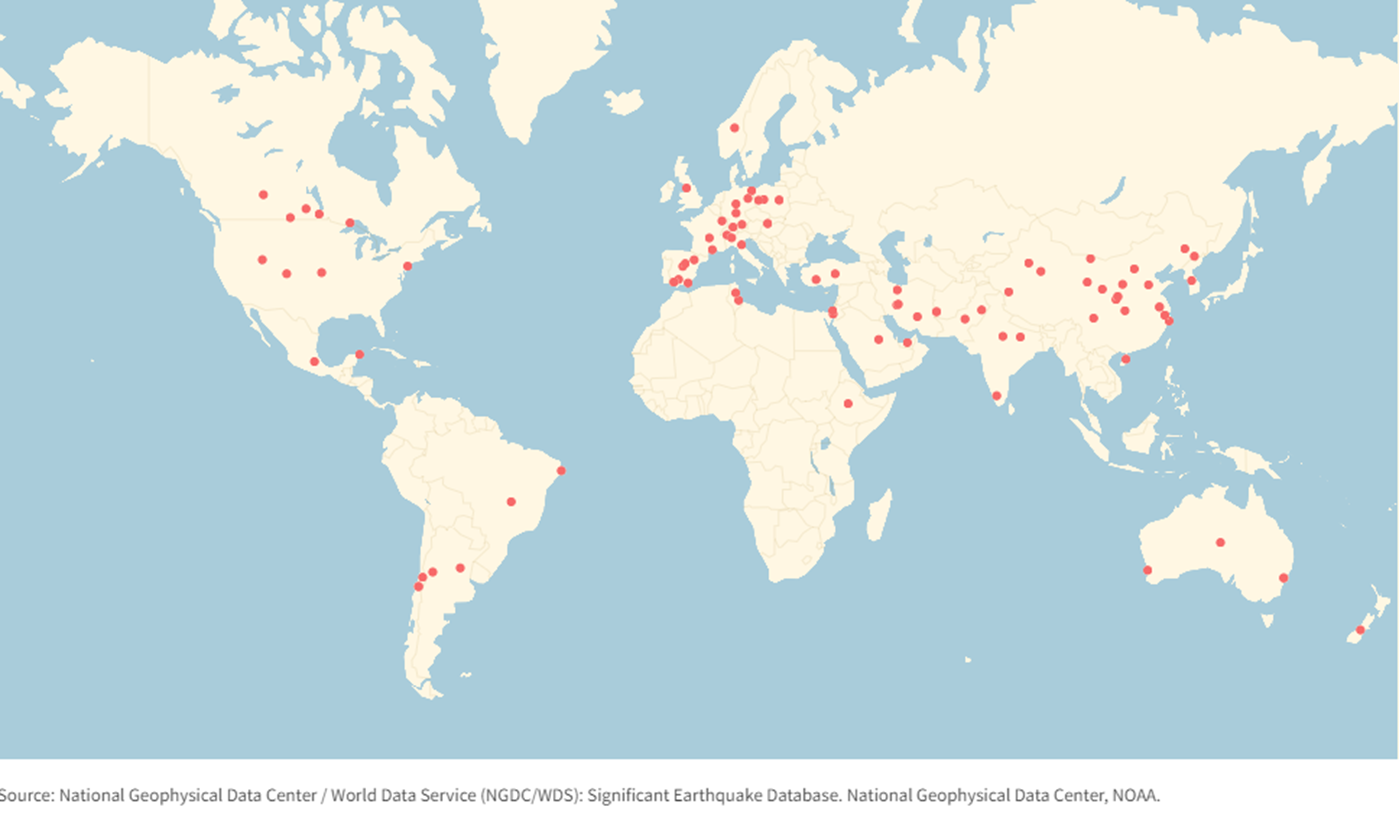


**Fig S2. Locations of all studies used in our meta-analysis.**


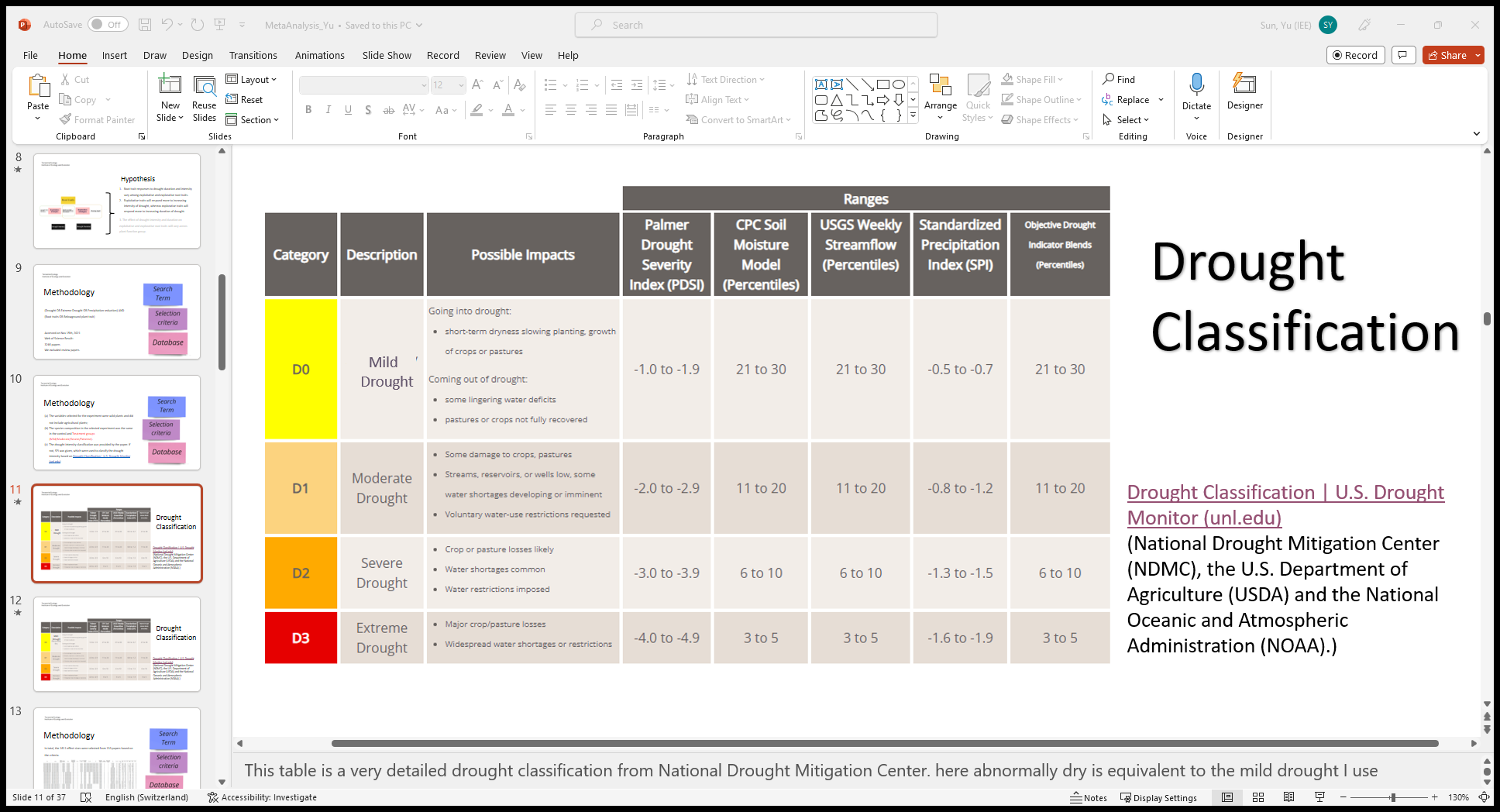


**Fig S3. Drought Classification (National Drought Mitigation Center (NDMC), the U.S. Department of Agriculture (USDA) and the National Oceanic and Atmospheric Administration (NOAA).)**
